# Unbiased estimation for response adaptive clinical trials

**DOI:** 10.1177/0962280215597716

**Published:** 2015-08-11

**Authors:** Jack Bowden, Lorenzo Trippa

**Affiliations:** 1 MRC Integrative Epidemiology Unit, University of Bristol, Bristol, UK; 2 MRC Biostatistics Unit, Cambridge, UK; 3 Dana-Farber Cancer Institute, Boston, USA

**Keywords:** Clinical trial, adaptive randomization, bias adjusted estimation, Horvitz-Thompson estimator, inverse probability weighting, Rao-Blackwellization

## Abstract

Bayesian adaptive trials have the defining feature that the probability of randomization to a particular treatment arm can change as information becomes available as to its true worth. However, there is still a general reluctance to implement such designs in many clinical settings. One area of concern is that their frequentist operating characteristics are poor or, at least, poorly understood. We investigate the bias induced in the maximum likelihood estimate of a response probability parameter, *p*, for binary outcome by the process of adaptive randomization. We discover that it is small in magnitude and, under mild assumptions, can only be negative – causing one’s estimate to be closer to zero on average than the truth. A simple unbiased estimator for *p* is obtained, but it is shown to have a large mean squared error. Two approaches are therefore explored to improve its precision based on inverse probability weighting and Rao–Blackwellization. We illustrate these estimation strategies using two well-known designs from the literature.

## 1 Introduction

From the emergence of clinical trials in medical research in the middle of the 20th century to the present day, a common paradigm has dominated the design and analysis of clinical trials as a means to improving patient care. Usually two treatments are assessed, one being the standard therapy and the other being an experimental treatment, that may offer an improvement in an appropriate patient outcome. The size of the trial is fixed in advance and analysis performed only at its conclusion. Patients entering the trial are randomized with a fixed probability (usually 1/2 for efficiency reasons) to either the experimental or control arm. The advantages of this sort of design are many: it is simple to calculate how many patients are needed to achieve a desired power for rejecting a given null hypothesis; by fixing the timing of the analysis the type I error rate can also be strictly controlled; randomization ensures (asymptotically) that patients on each of the treatment arms will be balanced with respect to all potential known and unknown confounding variables, except the treatment they took. This provides a solid basis for attributing, in a causal sense, the difference in outcomes between the two groups at the end of the trial to the treatment, and for unbiasedly estimating this difference using standard methods.

Over the last 20–30 years, modifications to the standard design have been proposed and accepted as a viable alternative within clinical research, although their use is still the exception rather than the rule. Group sequential designs^1,2^ introduced the idea of multiple analyses at fixed time points during its course, thus enabling early stopping when definitive evidence exists. Adaptive designs^3–5^ have added additional flexibility to the way in which mid-course data can be used to influence a study’s future scope. However, the above approaches still share a common characteristic: whilst it is ongoing, a fixed allocation probability is used for all arms still active in the trial. We subsequently refer to this as ‘fixed randomization’ (FR).

By contrast, the MD Anderson cancer centre (and others) has pioneered the use of an approach to clinical trials incorporating adaptive randomization (AR) as a fundamental component.^6,7^ The basic premise of AR is to use the accumulating evidence on the performance of all treatments to decide how to allocate future patients in the trial.^8^ They are becoming an increasingly popular vehicle for identifying effective patient-specific treatments in the new era of stratified medicine.^9^

There has been a weariness among the medical community to embrace designs incorporating AR, due to concerns over their frequentist properties^10^ – such as bias and type I error inflation – their inferiority to a comparable FR designs,^11^ and even their ethical validity.^12^ Indeed, recent Food and Drug Administration (FDA) guidance for the pharmaceutical industry cautions against the use of AR and recommends that operating characteristics of an AR design should be explored and understood before implementation^13^. In this paper we examine the affect that data-adaptive allocation has on the estimate of the true response probability, *p*, parameterising a binary outcome. We show that the bias induced in the maximum likelihood estimator (MLE) for *p* by AR is generally small and towards zero. A simple formula is derived that explains this phenomenon and a simple unbiased estimate for *p* is developed that is closely related to the Horvitz–Thompson (HT) estimator from survey sampling theory.^14^ Unfortunately, this estimator can have a large mean squared error (MSE) and even give estimates that lie outside the parameter space. Two strategies are proposed to improve the unbiased estimator’s precision. The first makes use of inverse probability of treatment weighting, as is commonly applied in the causal inference literature (see for example Hernan et al.^15^). The second approach involves the process of ‘Rao–Blackwellization’, a procedure commonly applied in sequential and adaptive trials.^16,17^

In Section 2 we define our notation and review the process of AR from a statistical perspective, in particular, its impact on the likelihood function. In Section 3 we investigate the small sample bias in the MLE induced by AR and compare this to the bias induced by other types of design adaptations. In Section 4 bias adjusted estimation is explored and in Section 5 we apply our bias adjustment strategies to a recent Bayesian adaptive design discussed in Trippa et al.^18^ Concluding remarks are made in Section 6 and possible avenues of future research are also discussed.

## 2 Data-adaptive allocation and the MLE

We assume that a randomized clinical trial is to be conducted on *n* patients with *K* treatment arms, the treatments being assessed via a binary response. The outcome for patient *i* given assignment to treatment *k*, *Y_ik_*, follows a Bernoulli distribution with parameter *p_k_*. Let *δ_ik_* be a binary variable equalling 1 if patient *i* is assigned to treatment *k* and zero otherwise, and let $\delta_{i}$ = ($\delta_{i1},\ldots$,*δ_ik_*). Define Δ*_i_* ∈ {1,…,K} as the treatment patient *i* is actually assigned to, so that $\delta_{i\Delta_{i}}\equiv$ 1. For simplicity, assume that patient responses are immediate, so that patient *i*’s response is known before patient *i* + 1 is recruited. The total information in the trial before patient *i* is recruited is the 2×(i-1) matrix:

(1) $$d_{i-1}=\left(\begin{array}{ccc} \Delta_{1} & \ldots & \Delta_{i-1}\\ y_{1,\Delta_{1}} & \ldots & y_{i-1,\Delta_{i-1}} \end{array}\right)$$

and let *d_n_* be the complete data on treatment assignments and outcomes at the end of the trial. Further let $n(k)=\sum_{i=1}^{n}\delta_{\mathrm{ik}}$ be the number of patients assigned to treatment *k* and $\sum_{i=1}^{n}\delta_{\mathrm{ik}}y_{\mathrm{ik}}$ be the number of responders to treatment *k*. Note that, whilst *n*(*k*) is random, the marginal total, $\sum_{k=1}^{K}n(k)$ = *n*, is fixed.

**Figure 1.**
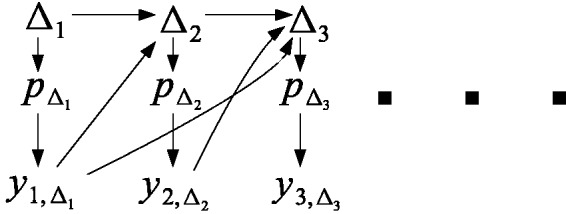
Illustrative diagram of data on the first three patients entered into a clinical trial using adaptive randomization.

In a trial that utilises AR, patient *i*’s allocation, Δ*_i_*, can be chosen to depend on all or part of *d_i_*_–1_, that is, on all previous patient randomization and outcome data. For illustration, Figure 1 depicts the dependence structure governing the randomization and outcome data for the first three patients recruited into a trial using AR. Here, $p_{\Delta_{i}}$ represents the true response parameter for treatment Δ*_i_*. Since Δ*_i_* is a random variable, so is $p_{\Delta_{i}}$. From Figure 1, we see that patient two’s randomization (Δ_2_) is allowed to depend on patient one’s outcome $y_{1,\Delta_{1}}$ and patient one’s randomization, Δ_1_ – both directly and indirectly (through $p_{\Delta_{1}}$). However, once $d_{1}=(\Delta_{1},y_{1,\Delta_{1}})$ has influenced the value of $\Delta_{2},y_{2,\Delta_{2}}$ is conditionally independent of *d*_1_, given Δ_2_. This statement clearly generalizes to the randomization and outcome of the *i*th patient given *d_i_*_–1_. We can therefore factorize the likelihood function for the parameter vector $p=(p_{1},\ldots ,p_{K})$ at the end of the trial as

(2) $$L(p|d_{n})=\overset{n}{\underset{i=1}{\Pi }}f_{p}(y_{i,\Delta_{i}}|\Delta_{i})f(\Delta_{i}|\Delta_{1},y_{1,\Delta_{1}},\Delta_{2},y_{2,\Delta_{2}}\ldots ,\Delta_{i-1},y_{i-1,\Delta_{i-1}})=\overset{n}{\underset{i=1}{\Pi }}f_{p}(y_{i,\Delta_{i}}|\Delta_{i})f(\Delta_{i}|d_{i-1})=\overset{n}{\underset{i=1}{\Pi }}f_{p}(y_{i,\Delta_{i}}|\Delta_{i})\pi_{i,\Delta_{i}}$$

The first component is the probability under ***p*** of observing patient *i*’s outcome given randomization to treatment Δ*_i_*. The second component, $\pi_{i,\Delta_{i}}$, is the probability that patient *i* was randomized to treatment Δ*_i_* conditional on previous data $d_{i-1}=(\Delta_{1},Y_{1,\Delta_{1}},\Delta_{2},Y_{2,\Delta_{2}}\ldots ,\Delta_{i-1},Y_{i-1,\Delta_{i-1}})$. Since this is a known function of the data it does not depend on the parameter vector ***p***. From standard likelihood theory (see for example Boos and Stefanski^19^), $\pi_{i,\Delta_{i}}$ can be removed from equation (2) when calculating the maximum likelihood estimate (MLE). This fact is obvious in a standard trial design using FR, where all *n* patients are randomized with equal probability to one of *K* arms. In this case there is clearly no information about the parameters in $\pi_{i,\Delta_{i}}$ – it takes the constant value 1/*K*. However, as we have shown above, the same is true within the AR framework described above. Removing $\pi_{i,\Delta_{i}}$ from the likelihood, and using the fact that the patient outcomes are independently Bernoulli distributed, expression (2) reduces to

$$L(p|d_{n})\propto \overset{K}{\underset{k=1}{\Pi }}p_{k}^{\sum_{i=1}^{n}\delta_{\mathrm{ik}}y_{\mathrm{ik}}}(1-p_{k})\sum_{i=1}^{n}\delta_{\mathrm{ik}}(1-y_{\mathrm{ik}})$$

and the MLE for *p_k_*, k=1,…,K is simply

(3) p^k=∑i=1nδikyikn(k)

Melfi and Page^20^ clarified that, in order for the MLE in equation (3) to be strongly consistent, there must exist a non-zero probability of being allocated to treatment *k* during the trial, as the trial size tends to infinity.

### 2.1 Case study: The Randomized Play the Winner rule

Wei and Durham^21^ proposed a randomized extension to Zelen’s original deterministic ’play-the-winner’ allocation rule^22^ for a two arm clinical trial, which serves as a useful toy example. The basic design works as follows: a hypothetical urn is imagined with one ball labelled ‘*T*_1_’ and another labelled ‘*T*_2_’, representing treatments one and two, respectively. A ball of type *T_k_* is picked at random from the urn, patient 1 is assigned to *T_k_* and the ball is returned to the urn. If patient 1 subsequently experiences a success, then another ball of type *T_k_* is added to the urn. However, if the patient experiences a failure, a ball of the alternative type is added instead. Thus, when a ball is picked at random from the urn to decide on the allocation of patient 2, they either have a 1/3 or a 2/3 chance of receiving treatment *k*. This procedure continues up until the planned end of the study and skews the treatment allocation towards the treatment that is performing the best. The probability that patient *i* is assigned to treatment *T*_1_ (so that Δ*_i_*  =  1) is simply the proportion of *T*_1_ balls in the urn after *i* – 1 patients, and is given by

$$\pi_{i,1}=\frac{1+\sum_{j=1}^{i-1}\delta_{j1}y_{j1}+\delta_{j2}(1-y_{j2})}{i+1}$$

The probability of randomization to *T*_2_, $\pi_{i,2}=1-\pi_{i,1}$. We will refer to this as the ‘randomized play-the-winner’ (RPW) design. When we implement the RPW design in later sections, we introduce a small modification whereby the first two patients are always split between arm 1 and 2. This guarantees that the estimates for *p*_1_ and *p*_2_ will always be defined but does not materially change its operating characteristics otherwise.

## 3 The bias of the MLE

Whilst AR leaves the asymptotic properties of the MLE intact, this does not give any indication as to its finite sample properties in a real trial context, in particular its bias. Without loss of generality, consider estimation of a single treatment’s parameter *p_k_*. We start by noting that

(4) E[n(k)p^k+(n-n(k))pk]=E[n(k)p^k]+E[(n-n(k))pk]

(5) =E[n(k)(p^k-pk)]+npk=npk

since

E[n(k)(p^k-pk)]=E[n(k)(∑i=1nδikyikn(k)-pk)]=0

From the standard definition for the covariance of two random variables, and making use of equations (4) and (5), we can write

Cov[n(k),p^k]+E[n(k)]E[p^k]+E[(n-n(k))pk]=npk

Further rearrangement and cancellation yield

(6) Cov[n(k),p^k]E[n(k)]=pk-E[p^k]=-Bias(p^k)

Equation (6) gives a simple characterisation of the bias. It is zero when *n*(*k*) is independent of p^k and increases in magnitude as they become more dependent. The bias is a decreasing function of *E*[*n*(*k*)] and will be opposite to the sign of their covariance. FR guarantees a zero covariance (and hence zero bias) since *E*[*n*(*k*)] = 1/*K* is a constant. However, in the common case where AR is used to direct more patients towards treatments that appear to work well, *n*(*k*) will be non-constant and positively correlated with p^k.

The bias induced by AR can be explained heuristically. Suppose that, in the early part of a clinical trial using AR, a treatment’s true effect is overestimated because an unusually high number of patients experience a positive response. In this case, AR will assign more patients to receive the treatment in the latter stages of the trial. The ‘random high’ in the treatment effect estimate then has the scope to regress back down towards its true value, the final MLE. However, suppose instead that in the early part of a trial a treatment’s effect is underestimated by chance. In this case fewer patients are assigned to receive the treatment in the latter stages and the treatment effect estimate does not have the same ability to regress back up towards its true value. This asymmetry creates a positive covariance between the MLE and its sample size and hence a negative bias.

**Figure 2.**
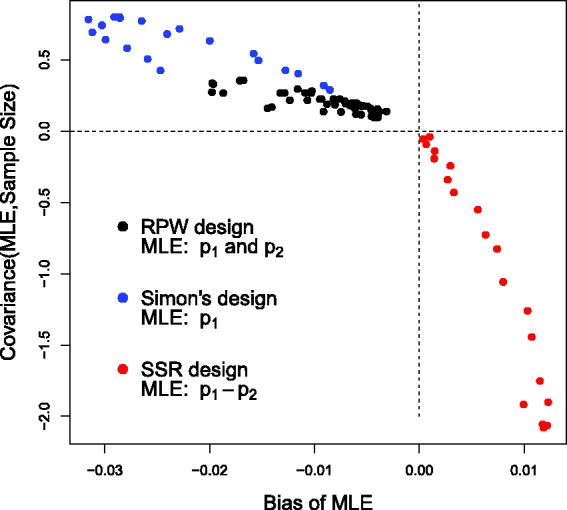
Bias of the MLE versus its covariance with the overall sample size for under three different trial designs. RPW: randomized play the winner, SSR: sample size re-estimation.

The black dots in Figure 2 show the bias induced in the response probability estimates for p^k as a function of Cov(n(k),p^k) under the RPW design with *n*  =  25 patients. Each dot represents a different parameter constellation in the region (*p*_1_,*p*_2_) ∈ (0.2,0.8). As equation (6) suggests, positive covariance implies negative bias in the MLE, and vice versa.

### 3.1 Further examples

Bauer et al.^23^ noted that a similar mechanism acts to induce a negative bias in a traditional sequential trial with early stopping. For example, consider a Simon’s two-stage single arm trial measuring a treatment’s response with respect to a binary outcome.^24^ Assume that 13 patients are initially allocated a treatment, with the trial continuing to full enrolment (34 patients) only if four or more patients are ‘responders’ at stage 1. If there are nine or more responders at the end of the trial the null hypothesis $H_{0}:p\leq 0.2$ can be rejected with a type I error rate of 10%.^25^ The number of patients allocated to treatment 1, *n*(1), is therefore a random variable:

$$n(1)=\{\begin{array}{cc} 13 & \text{if }\sum_{i=1}^{13}y_{i1}\leq =3\\ 34 & \text{Otherwise} \end{array}$$

The blue dots in Figure 2 show the bias in the estimates for the treatment’s response probability, *p*_1_, as the true value is varied between 0.2 and 0.4. Again, when large estimates go hand-in-hand with large sample sizes then a negative bias necessarily follows.

A much discussed adaptive design strategy exists that can induce exactly the opposite effect – that is a negative covariance between the MLE and the sample size it is based on (and therefore a positive bias). Consider a two-arm, two-stage trial that incorporates unblinded sample size re-estimation.^4,26,27^ In stage 1, 100 patients are initially allocated to each arm (using FR) and the MLE for the response parameters are estimated as p^1 and p^2. From Li et al.,^26^ we calculate the estimated standardised mean difference θ^ and stage 1 test statistic *z*_1_ via the variance stabilising transformation:

$$\hat{\theta }=2[{\text{sin}}^{-1}(\sqrt{{\hat{p}}_{1}})-{\text{sin}}^{-1}(\sqrt{{\hat{p}}_{2}})],\;z_{1}=\hat{\theta }\sqrt{\frac{100}{2}}$$

Suppose that if *z*_1_ is less than 1 or greater than 2.74 then the trial is stopped for efficacy or futility. However, if *z*_1_ ∈ (1,2.74) then further patients are recruited in order to give an 80% probability of rejecting the null hypothesis $H_{0}:\theta \leq 0$ at the alternative θ=θ^ with an unconditional type I error rate of 5%. This is similar to design 1 explored in Bowden and Mander.^27^ Making use of the Ceiling function ‘⌈.⌉’, the number of patients allocated to each arm (*n*(1) and *n*(2)) is again a random variable:

$$n(1)=n(2)=\{\begin{array}{cc} 100 & \text{if }z_{1}\notin (1,2.74)\\ \left\lceil \left[\frac{{\left(1.92+0.84\right)}^{2}}{z_{1}^{2}}-1\right]100\right\rceil & \text{Otherwise} \end{array}$$

The red dots in Figure 2 show the bias induced in the MLE for p1-p2,p^1-p^2, versus its covariance with the second stage sample size when *p*_1_ ∈ (0.45,0.65) and *p*_2_ is fixed at 0.3. Since small interim estimates of $p_{1}-p_{2}$ (with a corresponding *z*_1_ close to 1) are associated with large overall sample sizes, and large interim estimates (with a corresponding *z*_1_ close to 2.74) are associated with small sample sizes, the aforementioned covariance is negative and the bias is therefore positive.

## 4 Bias adjusted estimation

### 4.1 Simple bias adjustment

A simple bias-corrected estimate for *p_k_* exists that utilises $\pi_{i,\Delta_{i}}$ (when $\Delta_{i}=k$), namely

(7) p^k,HT=1n∑i=1nδikyikπi,k

p^k,HT bares strong resemblance to the HT estimator used extensively in survey methodology to correct for stratified sampling.^14^ It is not strictly identical because the $\pi_{i,k}$’s are not independent in equation (7). However, for ease we will refer to it as the HT estimator. We can see that it is indeed unbiased, because

$$E(\frac{1}{n}\sum_{i=1}^{n}\frac{\delta_{\mathrm{ik}}Y_{\mathrm{ik}}}{\pi_{i,k}})=\frac{1}{n}\sum_{i=1}^{n}E[E(\frac{\delta_{\mathrm{ik}}Y_{\mathrm{ik}}}{\pi_{i,k}}|d_{i-1})]$$

Since *δ_ik_* is independent of *Y_ik_* given *d_i_*_–1_ this can be written as

$$\frac{1}{n}\sum_{i=1}^{n}E[Y_{\mathrm{ik}}(\frac{E[\delta_{\mathrm{ik}}|d_{i-1}]}{\pi_{i,k}})]=\frac{1}{n}\sum_{i=1}^{n}E[Y_{\mathrm{ik}}]=p_{k}$$

Although unbiased, p^k,HT can have a large variance, in part because it can be larger than 1, which is clearly nonsensical. Of course, if we crudely constrain it to be less than 1 its unbiasedness is not maintained. We are therefore interested in alternative estimation strategies that improve upon p^k,HT, by naturally shrinking it to be within the unit interval and reducing its variance. A simple estimator that achieves this is a ‘normalised’ version of p^k,HT:

(8) p^k,IPW=np^k,HT∑i=1nδikπi,k

This can be thought of as the inverse probability (of treatment) weighted (IPW) estimate that is commonly used for causal inference of observational data^15^ and is the solution to the estimating equation

$$\sum_{i=1}^{n}\frac{\delta_{\mathrm{ik}}}{\pi_{i,k}}(y_{\mathrm{ik}}-p_{k})=0$$

It is not surprising that the IPW estimate appears a perfect fit for this context, given the similarity of the dependence structure present in trial data *d_n_* (and shown in Figure 1) to the phenomenon of time-varying confounding. The IPW estimate (8) is not unbiased for finite sized trials but is at least constrained to be in (0,1). Since the $\pi_{i,\Delta_{i}}$ terms are known exactly (one could not implement AR without them) the HT and IPW estimators are as trivial to calculate as the MLE.

**Figure 3.**
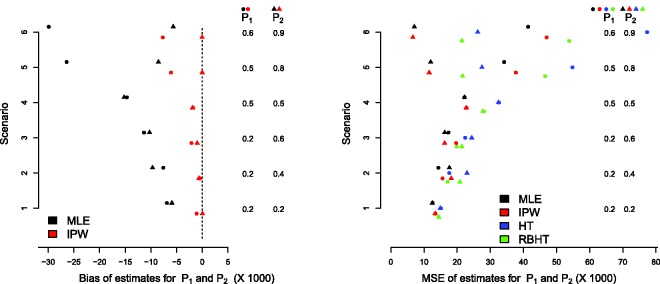
Left: bias of the MLE and IPW estimators. Right: MSE of the MLE, IPW, HT and RBHT estimators.

Figure 3 (left) shows the bias of the MLE and IPW estimators for the RPW design with *n*  =  25 patients. The bias of the HT estimate is not shown because it is zero (our simulations confirm this). Six different parameter combinations for ***p*** = (*p*_1_, *p*_2_) are considered – see the vertical columns in Figure 3 (left) – where the values of *p*_1_ and *p*_2_ and the bias in their estimates are denoted by circles and triangles, respectively. The results are the average of 50,000 simulations. Under RPW allocation, the MLE for p^k is always negatively biased. The bias is largest for the treatment with the smallest true effect size, and grows as the difference between the best and worst treatment increases. Although only the HT estimator is unbiased, the bias of the IPW estimator is essentially negligible for scenarios 1–4. However, for scenarios 5 and 6, a small negative bias is present. Figure 3 (right) compares the MSE of the MLE, IPW and HT estimators across the six scenarios (the RBHT estimator is discussed in the next section). Across all scenarios, the IPW estimator is shown to have a comparable MSE to the MLE. Predictably, the HT estimator has the largest MSE.

### 4.2 Rao–Blackwellization

A second option for improving the HT estimator is to use Rao–Blackwellization. Define the sufficient statistic, *S_n_*, for the parameter vector ***p*** to be the number of patients assigned to each arm, *n*(*k*), and the number of responders in each arm, $\sum_{i=1}^{n}\delta_{\mathrm{ik}}y_{\mathrm{ik}}$, for *k* = 1, … , *K*. Let the value of the sufficient statistic for the observed data be denoted by *s_n_*. Now define the set Ω* containing all possible trial realisations with sufficient statistics equal to *s_n_*, that is:

$$\Omega *=\{d_{n}^{*}:n*(k)=n(k),\sum_{i=1}^{n}\delta_{\mathrm{ik}}^{*}y_{\mathrm{ik}}^{*}=\sum_{i=1}^{n}\delta_{\mathrm{ik}}y_{\mathrm{ik}},k=1,\ldots ,K\}$$

To generate elements of Ω* we refer back to the data matrix in formula (1) and note that any permutation of the order of columns constituting matrix *d_n_* leaves *s_n_* unchanged and will therefore be in Ω*. However, at the same time, column permutations are the only transformation of *d_n_* allowed, since materially changing any individual element of *d_n_* will alter *s_n_*. In order to compute the Rao–Blackwellized Horvitz–Thompson (RBHT) estimator E(p^HT|sn) we need to integrate the HT estimate over Ω* as follows:

(9) p^RBHT=E(p^HT|sn)=∑Ω*p^HT(dn*)L(dn*;p)∑Ω*L(dn*;p)

where $L(d_{n}^{*};p)$ is the likelihood of observing data $d_{n}^{*}$ (a permutation of *d_n_*), under the true unknown parameters ***p***, as given in equation (2). The change of notation is deliberate, and for the reasons explained below. Since the cardinality of Ω* increases rapidly with the overall sample size, direct integration becomes unfeasible in most realistic settings and a Monte Carlo approximation is necessary. In order to achieve this end, we first note that ratio $\frac{L(d_{n}^{*};p)}{\sum_{\Omega *}L(d_{n}^{*};p)}$ in equation (9) remains constant across all values of ***p***. To see this, we rewrite $L(d_{n}^{*};p)$ as

(10) $$\overset{n}{\underset{i=1}{\Pi }}f_{p}(y_{i,\Delta_{i}}|\Delta_{i})\overset{n}{\underset{i=1}{\Pi }}\pi_{i,\Delta_{i}}^{*}$$

The first term is the likelihood of the data given treatment assignment; this product is constant for any permutation of *d_n_*, $d_{n}^{*}$, in Ω* (it does not matter what order the *n* terms are multiplied together). However, the second component of the likelihood does change under permutation, and so we write $\pi_{i,\Delta_{i}}^{*}$ to denote the probability that the *i*’th patient (in the permuted data set $d_{n}^{*}$, not the original data set *d_n_*) would have been randomized to treatment Δ*_i_* in $d_{n}^{*}$. We therefore have that

(11) $$L(d_{n}^{*};p)\propto \overset{n}{\underset{i=1}{\Pi }}\pi_{i,\Delta_{i}}^{*}$$

Given uniformly sampled trial realisations $d_{n}^{*}(j);\;j=1,\ldots ,M$, we can therefore approximate expectation (9) as:

(12) p^RBHT≈∑j=1Mp^HT(dn*(j))Π*(j)∑j=1MΠ*(j)

where Π*(j) = $\overset{n}{\underset{i=1}{\Pi }}\pi_{i,\Delta_{i}}^{*}$ under $d_{n}^{*}(j)$. Although this looks a simple and attractive procedure, most random permutations of the data will have an extremely small likelihood of occurrence, and equation (9) will generally be dominated by a small number of trial realisations clustered around the observed value, *d_n_*. An alternative Monte-Carlo approach consistent with this fact is to sample trial realisations ($d_{n}^{*}$’s) from the conditional distribution of *d_n_* given *s_n_* and obtain an approximation to equation (9) via an unweighted average:

(13) p^RBHT≈∑j=1Mp^HT(dn*(j))M

**Figure 4.**
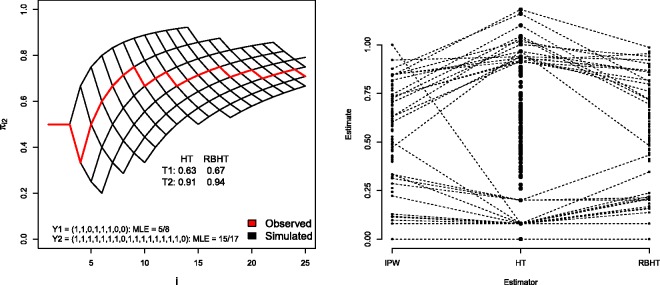
Left: $\pi_{i2}$ for single trial realisation under Scenario 6. Right: IPW, HT and RBHT estimates for *p*_1_ for 100 trial realisations under Scenario 6. Trial simulations giving rise to HT estimates above 0.8 and below 0.2 are linked to their respective IPW and RBHT estimates by dotted lines, in order to highlight the additional benefit that inverse probability weighting and Rao–Blackwellization provides.

For the RPW case study in Section 2.1, which contains only two arms and 25 patients, one can simply generate trial realisations from ($d_{25}|s_{25}$) by drawing *d*_25_’s unconditionally, and saving those with sufficient statistics equal to *s*_25_. A simple way to do this is to specify a value for the parameter vector ***p*** and, whilst any choice is valid, it is both natural and efficient to use the MLE. For illustration we consider a single trial realisation of the RPW design, simulated under scenario 6. In this case eight patients are randomized to treatment 1 (with 5 responders) and 17 to treatment 2 (with 15 responders), which defines *s*_25_. The red line in Figure 4 (left) shows the value of $\pi_{i2}$ for *i* = 1, …, 25. It starts at 1/2 (by design) and after 10 patients or so it stabilises at around 70%.

The MLE p^ = (5/8,15/17). The grid of black lines in Figure 4 (left) shows all realised values of $\pi_{i,2}$ over 500 simulated trials, $d_{25}^{*}(1),\ldots ,d_{25}^{*}(500)$. The HT and RBHT estimates for treatment 1 and 2 are also shown, the latter being the average of the HT estimates obtained from the 500 simulated trials. Figure 4 (right) shows the IPW, HT and RBHT estimates for *p*_1_, obtained across 100 trial realisations, again under scenario 6 (*p*_1_  =  0.6). Values above 0.8 and below 0.2 are linked to highlight that the IPW and RBHT estimators both constrain the HT estimate to be within (0,1) and shrink its variance. Figure 3 (right) in Section 4.1 shows RBHT estimator’s MSE compared to the other estimators for the RPW design. Its MSE is normally half way between that of the HT and IPW estimators. In summary, for the RPW design, the simple IPW estimator performs perfectly adequately, and there appears to be nothing gained in applying a more complex form of bias correction.

#### 4.2.1 A Metropolis–Hastings algorithm for the RBHT estimator

As the number of patients and number of arms in a trial increase, generating Monte Carlo draws from the conditional distribution $\left(d_{n}|s_{n}\right)$ by specifying a value for ***p*** also becomes infeasible, since the probability a random draw satisfying the condition is too small. We therefore propose a Monte Carlo Markov Chain approach to calculate the RBHT estimate. Now we need to construct an irreducible Markov Chain $d_{n}^{*}(j);\;j=1,\ldots ,M$, over the space of the $d_{n}^{*}$ sequences with stationary distribution ($d_{n}|s_{n}$). If possible, standard ergodicity arguments guarantee that the above Monte Carlo average of the $d_{n}^{*}(j)$’s in equation (13) converges with probability 1 to p^RBHT as *M* increases. Our construction of the Markov Chain is a direct application of the Metropolis–Hastings algorithm. We start from a $d_{n}^{*}(1)$ consistent with *s_n_* (for example $d_{n}^{*}(1)$ = *d_n_*) then at every j=1,…,M-1, a transition $d_{n}^{*}(j)\to d_{n}^{*}(j+1)$ is proposed. $d_{n}^{*}(j+1)$ is created by selecting randomly two integers (*i*_1_, *i*_2_) in {1,…,n}, and swapping the positions of the *i*_1_th and *i*_2_th columns of the 2×n matrix $d_{n}^{*}(j)$. At each step the usual Metropolis–Hastings rule accepts or rejects $d_{n}^{*}(j+1)$. The application of the Metropolis–Hastings rule requires one only to compute the probabilities on the right side of expression (11) and generates a Markov Chain with the desired stationary distribution. In the next section we apply the Metropolis–Hastings implementation of the RBHT estimator to a recent trial example.

## 5 An adaptive design for glioblastoma

Trippa et al.^18^ have recently proposed a Bayesian adaptive design for testing multiple experimental treatments in a controlled trial setting for patients with recurrent glioblastoma. Their motivation was to find a design that required fewer patients to identify an effective treatment compared to a standard multi-arm trial with equal randomization. Although the original cancer setting determined the use of a time-to-event outcome, the design approach can easily be transferred to the binary data setting. Indeed this has been the modus operandi for others to evaluate its operating characteristics^28–30^ and in the case of the latter, to offer a strong critique against its use. Let $\mathrm{Pr}(p_{k}-p_{0}>0|d_{i-1})$ be the posterior probability that treatment *k* (*k* = 1, …, *K*) provides a higher chance of success than the control (treatment *k* = 0), given all of the available data from patients 1, … , *i* – 1. The probability that patient *i* is randomized to treatment *K* under this scheme is^18^

$$\pi_{i,\Delta_{i}}=\{\begin{array}{cc} \frac{Pr{\left(p_{k}-p_{0}>0|d_{i-1}\right)}^{\gamma_{i}}}{\sum_{j=1}^{K}Pr{\left(p_{i}-p_{0}>0|d_{i-1}\right)}^{\gamma_{i}}} & \text{for }k\text{ in }1,...,K\\ \frac{1}{K}\text{exp}{\left\{\text{max}(n_{i-1,k},k=1,...K)-n_{i-1,0}\right\}}^{\eta_{i}} & \text{for }k=0 \end{array}$$

The term $n_{i-1,k}$ represents the number of patients who have been assigned to treatment arm *k* after *i* − 1 patients have been recruited into the trial. The *γ_i_* and *η_i_* terms are positive increasing functions of *i* which can be ‘tuned’ to fix the desired characteristics of the trial. The total sample size of the trial, *n*, is fixed as before. We refer the reader to Trippa et al.^18,31,32^ for the interpretation of the tuning parameters (*γ_i_*, *η_i_*) and extensions with composite outcomes models, including progression free survival and overall survival times. This AR procedure achieves two goals: To allocate more patients to the experimental arm which is performing the best (for the benefit of patients in the trial); to allocate a similar number of patients to the control arm as the best experimental arm (to protect the trial’s overall power).

**Figure 5.**
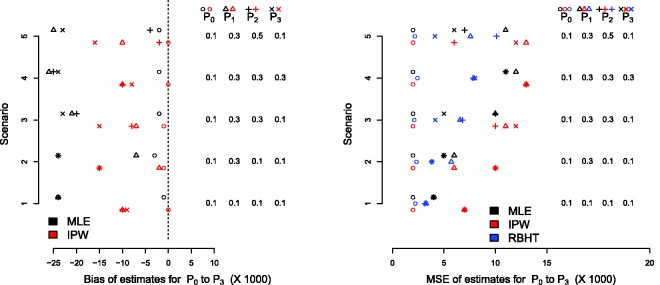
Bias (left) and MSE (right) for the glioblastoma case study.

Following Friedlin and Korn^30^ and Trippa et al.,^29^ 5000 trials of *n*  =  140 patients are simulated under this design with three active arms and one control arm. Uniform beta(1,1) priors were used to initialise the Bayesian analysis, so that the posterior distributions for each *p_k_* were also beta distributed. The allocation probabilities can then be calculated using a simple Monte Carlo approximation. The function’s *γ_i_* and *η_i_* were chosen to be 10(*i*/*n*)^1.75^ and 0.25(*i*/*n*), respectively. Figure 5 shows the bias and MSE of the MLE and IPW estimators under the five parameter constellations considered in Friedlin and Korn^30^ and Trippa et al.^29^ The RBHT estimate was calculated using the Metropolis–Hastings algorithm described in Section 4.2.1. We show its MSE only since it is unbiased (again, simulations confirm this) and plotting its estimated bias at zero obscures the other results.

**Figure 6.**
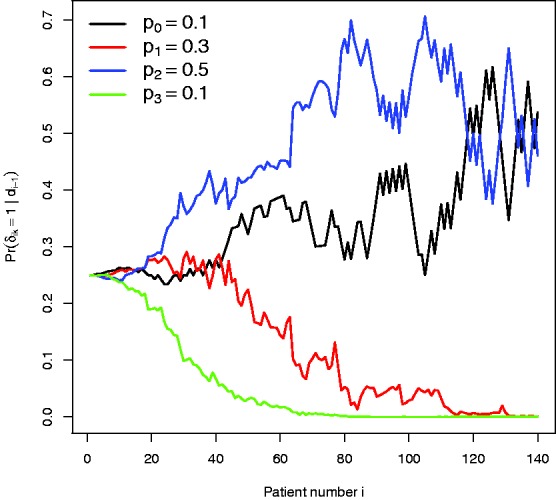
Randomization probabilities for a single trial realisation under parameter constellation 5.

By design, there is very little bias in the standard MLE for the control group across all scenarios. The MLE for the experimental treatments is, however, consistently negatively biased. The IPW estimate reduces the bias in the MLE, but its performance is not uniformly as impressive as for the RPW(1,1) design, especially for treatments with relatively small effect sizes. We suspect this is because the probability of randomization to a poorly performing treatment may approach zero, which makes the IPW estimator naturally unstable. Figure 6 illustrates this point for single trial realisation under scenario 5, where treatment 3 is significantly worse than the other experimental treatments. After 60 patients have been recruited the probability of allocation to arm three is essentially zero. Consequently, the IPW estimator generally has a larger MSE than the MLE, as seen in Figure 5 (right). The RBHT estimator perfectly removes the bias across all scenarios (results not shown). More importantly, it also has a smaller MSE than either the MLE or IPW estimators, making it attractive for this design.

## 6 Discussion

In this paper we have considered the effect that AR has on the estimation of a treatment’s true response rate within the context of a clinical trial. We have clarified that, whilst the simple proportion of total responders is still the MLE under AR, it will be biased in small samples (but not seriously so). We restricted our attention to the bias in each treatment parameter *p_k_* and not, for example, on treatment control response differences *p_k_*  −  *p*_0_. This was to explain the issue of bias in its most clear and general terms. Furthermore, we do not consider the issue of hypothesis testing, for which such differences would naturally form a basis. In the glioblastoma design, since the control group’s allocation probability was not dependent on its patient response rate, the MLE p^0 was essentially unbiased and therefore all of the bias in the difference p^k-p^0 is driven by p^k. However, when the control group is not treated differently and all arms in a trial are subject to the same AR scheme (as in the RPW example) the bias of the difference p^k-p^j can be either positive or negative, but it will generally be even smaller than the bias for p^k or p^j. This is because some of the negative bias will cancel out.

The HT estimator was shown to perfectly adjust for the bias in *p_k_*, but was not attractive to use in practice. Inverse probability weighting – commonly used in the causal inference and missing data literature – offered a simple means to improve the HT estimator. Its performance was poor, however, if the allocation probability weights were allowed to approach zero. IPW estimators can be improved via the use of ‘stabilised weights’,^15^ and it would be interesting to explore whether their use in this context.

Rao–Blackwellization was shown to offer the most comprehensive (but computationally intensive) means to improve the HT estimator. If *S_n_* is a complete and sufficient statistic for ***p*** then p^RBHT could claim to be the uniform minimum variance unbiased estimate (UMVUE) for ***p***. This technique has been suggested in the analysis of group sequential trials by Emerson and Fleming.^16^ In their case, the MLE for *p_k_* at the point of the first interim analysis (i.e. the first chance to stop the trial) is Rao–Blackwellized, since only information collected up to this point is unbiased. In our case, we are using a weighted average of the entire data. However, due to concerns about verifying the completeness of *S_n_*, we simply refer to it as a ‘Rao–Blackwellized’ estimator. By the Rao–Blackwell theorem, the RBHT estimate will be unbiased with a smaller variance than the HT estimator. The treatment control difference, *p_k_*  −  *p*_0_, is likely to be the primary outcome measure in a clinical trial. Since the RBHT estimator is calculated for the complete vector of response parameters, any linear combination of the RBHT parameter estimates (such as a treatment control difference) can be subsequently calculated (with unbiasedness maintained). This makes the RBHT estimator more widely applicable in practice than showcased here.

Another strategy for bias adjusted inference in group sequential trials, due to Whitehead^33^ and termed the bias-corrected MLE, is to find the vector ✓p satisfying: E(p^|✓p)=p^. In words, ✓p is the parameter constellation for which the expected value of the MLE (given trial data generated under ✓p) is equal to the observed MLE. In practice, the vector ✓p is estimated via an iterative process. It has been shown to perform well in group-sequential trials using FR^16,25^ and one could in theory apply it to this context. However, if one adopts a Monte-Carlo approach here too, then it is not attractive, because of the inherent difficulty in assessing the convergence of an iterative process containing a stochastic element. In contrast, whilst calculation of the RBHT estimate involves the generation of trial realisations, no such iteration is necessary.

Besides statistical bias, other types of biases have been highlighted as particularly problematic for trials utilising AR. These biases are very important but are beyond the scope of this paper. However, we now briefly allude to some obvious examples. The un-blinding of patient data mid-trial in order to implement AR can lead to so called ‘operational’ bias, if this information leaks to an individual who has the power and authority to misuse it.^34^ Another example occurs when the patients recruited into the trial systematically differ over time in their characteristics, and those characteristics are not independent of the treatment’s effect. When so called ‘patient drift’ exists, AR trials are again susceptible to bias and type I error rate inflation when FR trials are not.^35^ In this second case, however, bias adjusted estimation could potentially be extended to this setting by defining the randomization probability weights conditional on any measured time varying covariates.

A complete discussion as to the relative merits of AR versus FR is also beyond the scope of this paper. We refer the reader to the interesting discussions in Berry^36^ and Friedlin and Korn,^30^ that reach opposite conclusions. In recent editorial on the subject Thall et al.^37^ conclude that in early phase trials, where the goal is often to select the most promising dose from many candidates, AR holds sufficient promise to be considered as a design choice. However, they also discuss that in other trial settings, any benefit provided by an AR scheme is likely to be negated by the logistical complications of implementation. In a recent commentary article, Hey and Kimmelman^12^ appear to rule out the use of AR in two-arm trials, but echo the sentiments of Thall et al. in indicating its potential utility in multi-arm trial settings. In our investigation we also found the performance of the various bias adjusted estimators to be better in the multi-arm glioma trial context than the two arm RPW trial context. This is because multi-arm trials inherently put a stronger emphasis on treatment selection, which leads to more pronounced biases and hence the need for bias adjustment.

## References

[1] Pocock SJ Group sequential methods in the design and analysis of clinical trials. Biometrika 1976; 64: 191–199.

[2] Jennison C Turnbull BW Group sequential methods with applications to clinical trials, London: Chapman and Hall, 1999.

[3] Bauer P Kohne K Evaluations of experiments with adaptive interim analyses. Biometrics 1994; 50: 1029–1041. 7786985

[4] Proschan MA Hunsberger SA Designed extension of studies based on conditional power. Biometrics 1995; 51: 1315–1324. 8589224

[5] Cui L Hung HMJ Wang SJ Modification of sample size in group sequential clinical trials. Biometrics 1999; 55: 321–324. 11315017 10.1111/j.0006-341x.1999.00853.x

[6] Lee JJ Gu X Liu S Bayesian adaptive randomization designs for targeted agent development. Clin Trials 2010; 7: 584–596. 20571130 10.1177/1740774510373120 PMC5110207

[7] Alexander BM Wen PY Trippa L et al. Biomarker-based adaptive trials for patients with glioblastoma? lessons from i-spy 2. Neuro-oncology 2013; 15: 972–978. 23857706 10.1093/neuonc/not088 PMC3714161

[8] Berry DA Eick SG Adaptive assignment versus balanced randomization in clinical trials: a decision analysis. Stat Med 1995; 14: 231–246. 7724909 10.1002/sim.4780140302

[9] Barker AD Sigman CC Kelloff GJ et al. I-spy 2: an adaptive breast cancer trial design in the setting of neoadjuvant chemotherapy. Clin Pharmacol Ther 2009; 86: 97–100. 19440188 10.1038/clpt.2009.68

[10] Ventz S Trippa L Bayesian designs and the control of frequentist characteristics: a practical solution. Biometrics. 2015; 71: 218–226. 10.1111/biom.12226 25196832

[11] Korn EL Friedlin B Outcome-adaptive randomization: Is it useful? J Clin Oncol 2011; 29: 771–776. 21172882 10.1200/JCO.2010.31.1423 PMC3056658

[12] Hey SP Kimmelman J Are outcome adaptive allocation trials ethical? Clin Trials 2015; 12: 102–106. 25649106 10.1177/1740774514563583 PMC4482671

[13] FDA. Guidance for industry: adaptive design clinical trials for drugs and biologics. Technical report, US Food and Drug Administration, 2010.

[14] Horvitz DG Thompson DJ A generalization of sampling without replacement from a finite universe. J Am Stat Assoc 1952; 47: 663–685.

[15] Hernan MA Brumback BB Robins JM Estimating the causal effect of zidovudine on cd4 count with a marginal structural model for repeated measures. Stat Med 2002; 21: 1689–1709. 12111906 10.1002/sim.1144

[16] Emerson SS Fleming TR Parameter estimation following group sequential hypothesis testing. Biometrika 1990; 77: 875–892.

[17] Bowden J Glimm E Conditionally unbiased and near unbiased estimation of the selected treatment mean for multi-stage drop-the-losers trials. Biometrical J 2014; 56: 332–349. 10.1002/bimj.201200245 PMC4034592 24353149

[18] Trippa L Lee EQ Yen PY et al. Alexander. Bayesian adaptive trial design for patients with recurrent gliobastoma. J Clin Oncol 2012; 30: 3258–3263. 22649140 10.1200/JCO.2011.39.8420 PMC3434985

[19] Boos D Stefanski LA Essential statistical inference, New York: Springer, 2013.

[20] Melfi VE Page C Estimation after adaptive allocation. J Stat Plann Inference 2000; 87: 353–363.

[21] Wei LJ Durham S The randomized play-the-winner rule in medical trials. J Am Stat Assoc 1978; 85: 840–843.

[22] Zelen M Play-the-winner rule and the controlled clinical trial. J Am Stat Assoc 1969; 64: 131–146.

[23] Bauer P Koenig F Brannath W et al. Selection and bias – two hostile brothers. Stat Med 2010; 30: 1–11. 19844944 10.1002/sim.3716

[24] Simon R Optimal two-stage designs for phase II clinical trials. Controll Clin Trials 1989; 10: 110–110. 10.1016/0197-2456(89)90015-9 2702835

[25] Bowden J Wason JMS Identifying combined design and analysis procedures in two stage trials with a binary endpoint. Stat Med 2012; 31: 3874–3884. 22786815 10.1002/sim.5468 PMC3546375

[26] Li G Shih WJ Xie T A sample size adjustment procedure for clinical trials based on conditional power. Biostatistics 2002; 3: 277–287. 12933618 10.1093/biostatistics/3.2.277

[27] Bowden J Mander A A review and re-interpretation of a group sequential approach to sample size re-estimation in two stage trials. Pharm Stat 2014; 13: 163–172. 24692348 10.1002/pst.1613 PMC4288989

[28] Wason JMS Trippa L A comparison of Bayesian adaptive randomisation and multi-stage designs for multi-arm clinical trials. Stat Med 2014; 33: 2206–2221. 24421053 10.1002/sim.6086

[29] Trippa L Lee EQ Yen PY et al. Reply to b. Friedlin et al. J Clin Oncol 2013; 31: 970–971. 23565532 10.1200/JCO.2012.47.4403

[30] Friedlin B Korn EL Letter to the editor: adaptive randomisation versus interim monitoring. J Clin Oncol 2013; 31: 969–970. 23341515 10.1200/JCO.2012.45.0254

[31] Trippa L Wen PY Parmigiani G et al. Combining progression-free survival and overall survival as a novel composite endpoint for glioblastoma trials. Neuro-oncology 2015 17: 1106–1113. 10.1093/neuonc/nou345 PMC4490868 25568226

[32] Alexander BM Trippa L Progression-free survival: too much risk, not enough reward? Neuro-oncology 2014; 16: 615–616. 24733851 10.1093/neuonc/nou041 PMC3984564

[33] Whitehead J On the bias of the maximum likelihood estimate following a group sequential test. Biometrika 1986; 73: 573–581.

[34] Chow SC Chang M Adaptive design methods in clinical trials a review. Orphanet J Rare Dis 2008; 3: 11–11. 18454853 10.1186/1750-1172-3-11 PMC2422839

[35] Simon R Simon N Using randomization tests to preserve type I error with response-adaptive and covariate-adaptive randomization. Stat Probab Lett 2011; 81: 767–772. 21769160 10.1016/j.spl.2010.12.018 PMC3137591

[36] Berry DA Adaptive clinical trials: the promise and the caution. J Clin Oncol 2011; 29: 606–609. 21172875 10.1200/JCO.2010.32.2685

[37] Thall PF Fox P Wathen JK Some caveats for outcome adaptive randomization in clinical trials. In: Sverdlov O (ed). Modern adaptive randomized clinical trials: statistical, operational, and regulatory aspects. Randomization in clinical trials, Oxford: Taylor & Francis, 2015.

